# Genetic signatures of *AKT1* variants associated with worse COVID-19 outcomes – a multicentric observational study

**DOI:** 10.3389/fimmu.2024.1422349

**Published:** 2024-10-08

**Authors:** Ingrid Marins de Almeida, Bruna Ramos Tosta, Laiane da Cruz Pena, Hatilla dos Santos Silva, Fabiane S. Reis-Goes, Nívia N. Silva, João Victor Andrade Cruz, Mailane dos Anjos Silva, Jéssica Francisco de Araújo, Juliana Lopes Rodrigues, Gabriella Oliveira, Ricardo Gassmann Figueiredo, Sara Nunes Vaz, Iris Montaño-Castellón, Daniele Santana, Alex Torres, Fabyan Esberard de Lima Beltrão, Valdirene Leão Carneiro, Gubio Soares Campos, Carlos Brites, Vitor Fortuna, Camila Alexandrina Figueiredo, Soraya Castro Trindade, Helton Estrela Ramos, Ryan dos Santos Costa

**Affiliations:** ^1^ Laboratório de Imunofarmacologia e Biologia Molecular, Instituto de Ciências da Saúde, Universidade Federal da Bahia, Salvador, Brazil; ^2^ Laboratório de Imunologia e Biologia Molecular, Instituto de Ciências da Saúde, Universidade Federal da Bahia, Salvador, Brazil; ^3^ Laboratório de Análises Clínicas, Instituto Couto Maia, Salvador, Brazil; ^4^ Universidade Estadual de Feira de Santana, Feira de Santana, Brazil; ^5^ Hospital Universitário Professor Edgard Santos, Universidade Federal da Bahia, Salvador, Brazil; ^6^ Hospital Universitário Lauro Wanderley, Universidade Federal da Paraíba, João Pessoa, Brazil; ^7^ Departamento de Ciências da Vida, Universidade do Estado da Bahia, Salvador, Brazil; ^8^ Laboratório de Virologia, Instituto de Ciências da Saúde, Universidade Federal da Bahia, Salvador, Brazil; ^9^ Programa de Pós-Graduação em Processos Interativos de Órgãos e Sistema, Instituto de Saúde e Ciência, Universidade Federal da Bahia, Salvador, Brazil

**Keywords:** AKT1, COVID-19, severity, polymorphism, immunogenetics

## Abstract

**Introduction:**

The COVID-19, triggered by the SARS-CoV-2 virus, has varied clinical manifestations, ranging from mild cases to severe forms such as fatal pneumonia and acute respiratory distress syndrome (ARDS). Disease severity is influenced by an exacerbated immune response, characterized by high pro-inflammatory cytokine levels. Inhibition of AKT can potentially suppress pathological inflammation, cytokine storm and platelet activation associated with COVID-19. In this study, we aimed to investigate the rs2494746 and rs1130214 variants in the *AKT1* gene associated with severe COVID-19 outcomes.

**Methods:**

Peripheral blood samples and sociodemographic data from 508 individuals with COVID-19, measuring plasma cytokine concentrations using ELISA and genotyped the *AKT1* variants.

**Results:**

The rs2494746-C allele was associated with severity, ICU admission, and death from COVID-19. The C allele at rs1130214 was linked to increased TNF and D-dimer levels. Moreover, both variants exhibited an increased cumulative risk of disease severity, ICU admission, and mortality caused by COVID-19. In the predictive analysis, the rs2494746 obtained an accuracy of 71%, suggesting a high probability of the test determining the severity of the disease.

**Discussion:**

Our findings contribute to understanding the influence of the *AKT1* gene variants on the immunological damage in individuals infected with SARS-CoV-2.

## Introduction

COVID-19, caused by the SARS-CoV-2 virus, has emerged as a worldwide crisis. Transmitted mainly through the respiratory route, the virus infiltrates the host by binding to angiotensin-converting enzyme 2 (ACE2) receptors, found extensively in tissues and immune cells. This invasion can give rise to diverse clinical manifestations, ranging from mild cases to severe or critical forms that can lead to fatal outcomes (1).

Several studies have reported that immunopathological damage caused by highly elevated levels of pro-inflammatory cytokines produced by immune and epithelial cells can lead to fatal pneumonia and acute respiratory distress syndrome (ARDS) (2). As accumulating evidence indicates that severe COVID-19 is a multifaceted inflammatory disease affecting multiple systems, it’s also worth noting that comorbidities like hypertension, heart failure, cardiac arrhythmia, diabetes, kidney failure, and chronic lung disease, as well as factors such as older age and male gender, are linked to unfavorable clinical outcomes (3).

Host genetics may also be important in the severity of COVID-19. AKT, a serine/threonine protein kinase that has 3 isoforms (AKT1, AKT2, and AKT3), is part of the PI3K/AKT/mTOR pathway, a crucial cellular signaling pathway that regulates various cellular functions (4). A proteomics and transcriptomics study demonstrated that cells infected with SARS-CoV-2 showed increased AKT/mTOR pathway signaling. Inhibiting this pathway can significantly reduce virus production (5). The PI3K/AKT pathway regulates the generation and function of regulatory T cells (Tregs) (6). This suggests that increasing the population of regulatory T cells (Tregs) might promote lung recovery in patients diagnosed with acute respiratory distress syndrome (7).

Although hyperactivation of various immune system cellular components has been observed in patients with severe COVID-19, the host genetic factors determining susceptibility to disease severity are not entirely understood (8). Previous studies have shown that variants of the *AKT1* gene may be associated with other diseases (9–12).

Our studies previously indicated a possible implication of the AKT/mTOR pathway in severe manifestations of COVID-19. The association of the rs1057079 variant of the *MTOR* gene with severe and critical outcomes suggests a direct influence of this pathway on disease severity. Furthermore, the association between the rs2536 variant and elevated levels of IL-6 and mortality from COVID-19 suggests a possible role for this pathway in regulating the exacerbated inflammatory response characteristic of the cytokine storm observed in severe cases of the disease (13). These findings provide important insights into the potential impact of the AKT/mTOR pathway on the pathophysiology of COVID-19. Therefore, this study aimed to investigate the association between variants in the *AKT1* gene and worse outcomes of COVID-19.

## Materials and methods

### Study population

Five hundred-eight individuals with COVID-19, 216 mild cases and 292 severe cases, were recruited from April 2020 to April 2021, prior to the national vaccination period. Severe cases were recruited at Hospital Metropolitano Dom José Maria Pires (Paraíba, Brazil), Hospital EMEC (Empreendimentos Médico Cirúrgicos Ltda), and Hospital Couto Maia (Bahia, Brazil). Among the severe cases, 103 patients were admitted to the Intensive Care Unit (ICU), and 53 died. A non-probabilistic sample of mild subjects was recruited at the Primary Care Unit of the Professor Edgard Santos University Hospital - HUPES (Bahia, Brazil) and in the community.

Recruitment was conducted through three studies previously approved by the Human Research Ethics Committee of the Lauro Wanderley University Hospital (Paraíba, Brazil) under n°. 31562720.9.0000.5183, by the Research Ethics Committee of the State University of Feira de Santana (Bahia, Brazil) under n°. 30764720.1.0000.0053, and the Research Ethics Committee of HUPES (Bahia, Brazil) under no. 31748320.3.1001.5543.

### Diagnosis of COVID-19

Confirmation of infection was performed by quantitative reverse transcription polymerase chain reaction (RT-qPCR) testing for SARS-CoV-2 with samples from the respiratory tract (nasopharyngeal swab, airway aspiration, or sputum induction) or in cases of negative RT-qPCR, clinical, radiological diagnosis (ground-glass opacities, with or without consolidation, located close to visceral pleural surfaces and bilateral distribution - CO-RADS 5) and positive serological test (IgG for SARS-CoV-2) were used (14).

### Severity of COVID-19

The definition of severity within 48 hours after patient admission was established using two scoring systems: 1) the “Quick Sequential Organ Failure Assessment (qSOFA)” score, 2) the “National Early Warning Score 2 (NEW2)”, analyzing CT scans and application of the severity score (15) or accessing parameters such as pulse oxygen saturation less than 94%, respiratory rate persistently above 24 breaths per minute, or indication for hospital admission.

Intensive care unit (ICU) admission data and mortality were used for secondary analyses. The severe group was divided into critical patients - individuals admitted to the ICU, and non-critical patients – individuals not admitted to the ICU. Blood samples were collected before interventions or therapies that could interfere with cytokine results. We considered the mild form of the disease to be all individuals with SARS-CoV-2 infection and the absence of the aforementioned risk factors.

### DNA extraction and quantification

DNA extraction was performed from whole blood and buffy coat samples according to the standard protocol with the FlexiGene^®^ DNA kit (Qiagen GmbH, Hilden, Germany). DNA concentration and purity were estimated by spectrophotometry with the “NanoDrop™ Lite Spectrophotometer” (Thermo Fisher Scientific, Wilmington, DE, USA). We standardized at a 5 ng/μL concentration using nuclease-free water in a 96-well molecular biology plate and stored in a freezer at -20°C for later use.

### Selection and genotyping of SNPs

The SNPs rs1130214 and rs2494746 were selected for genotyping based on specific criteria. These criteria included minor allele frequency (MAF) between 0.05 and 0.5, functional impact on the gene assessed by RegulomeDb (http://regulomedb.org/), and previous associations with clinical conditions related to *AKT1* gene expression and an unfavorable COVID-19 prognosis, such as cancer, lung, viral, heart, and mental diseases, as indicated by Ensembl (http://www.ensembl.org/index.html) and the National Center for Biotechnology Information (NCBI) (http://www.ncbi.nlm.nih.gov/). Additionally, linkage disequilibrium with other variants was also considered, as assessed by HaploReg V4.1 (http://compbio.mit.edu/HaploReg).

The genotyping assay was performed with the GoTaq Probe qPCR Master Mix kit (Promega, WI) and the TaqMan^®^ SNP Genotyping Assays probe (Thermo Fisher Scientific, Waltham, MA, USA). RT-qPCR was performed on the QuantStudio ™ 12K Flex Real-Time PCR System (Applied Biosystems, Life Technologies, Carlsbad USA).

### Plasma concentration of cytokines

Plasma concentrations of cytokines were measured using the Human TNF alpha Uncoated ELISA, Human IL-6 Uncoated ELISA, and Human CCL2 (MCP-1) Uncoated ELISA (Invitrogen; Thermo Fisher Scientific, Waltham, MA, USA) according to the manufacturer’s protocol. We used the Multiskan GO Spectrophotometer (Thermo Fisher Scientific, Waltham, MA, USA) to read the absorbance at 450 nm. The kit’s detection limit was 4–500 pg/mL for TNF, 2–200 pg/mL for IL-6, and 7-1000 pg/mL for CCL2. A standard curve was generated with the optical density (OD) values, and to perform the comparative analysis, only samples with concentrations ≥ the lowest point of the curve of each cytokine kit were used.

### D-dimer

According to the manufacturer’s protocol, D-dimer plasma concentrations were assessed using chemiluminescence immunoassay (MAGLUMI-2000-PLUS, Shenzhen New Industries Biomedical Engineering Co., Shenzhen, China).

### Statistical analysis

Descriptive statistical analyses were performed on comparative groups, such as the chi-square test, Fisher’s exact test, and Mann-Whitney U test, using IBM^®^ SPSS^®^ Statistics 25 software (SPSS Inc., Illinois, USA). For quality control of genotyping data, the following filters were applied: genotyping rate < 0.90 and minor allele frequency (MAF) < 0.05. Association analyses for categorical variables were conducted using logistic regression with three genetic models of inheritance (additive, dominant, and recessive) in PLINK 1.9 software.

To estimate the cumulative contribution of risk alleles to outcomes, individuals were categorized into groups with no risk allele or with one risk allele (≤1, GG+AA; GG+AC; AA+CG), two alleles (GG+CC; CC+AA; CG+AC), three risk alleles (CG+CC; CC+AC), and four risk alleles (CC+CC). Logistic regression analyses for severity and critical outcomes were performed using R Statistical Software packages (v 4.1.1; R Core Team 2022). Both analyses were adjusted for defined covariate values, such as age, sex, hypertension, diabetes, and heart disease for the severity variable; age, sex, diabetes, and heart disease for the critical condition variable; and age and sex for the death variable.

For cytokine and D-dimer analyses, normality was initially assessed using the Kolmogorov-Smirnov test. Considering the non-parametric distribution of the data, an unpaired two-tailed Mann-Whitney U test was employed for comparisons between groups, and the data were presented as median and interquartile range. Survival analysis was conducted using the Kaplan-Meier method to plot survival curves for a subsample of severe cases, the log-rank test (Mantel-Cox) to compare the two survival curves, and the Hazard Ratio (Mantel-Haenszel) to measure the time at which individuals died. GraphPad Prism 8 statistical software (GraphPad Inc., San Diego, CA) was utilized for both analyses.

The prediction analysis involved conducting Cohen’s Kappa Test to assess the agreement between the variable of interest (disease severity) and the genotypes of the rs2494746 and rs1130214 variants, using IBM^®^ SPSS^®^ Statistics 25 software (SPSS Inc., Illinois, USA). After obtaining the test results, the following performance metrics were calculated: Accuracy (A/(A+B+C+D)), Sensitivity (A/(A+C)), Specificity (D/(B+D)), Positive Predictive Value (PPV) (A/(A+B)), and Negative Predictive Value (NPV) (D/(C+D)). The values A, B, C, and D, representing true positives, false negatives, false positives, and true negatives, respectively, were used in the calculations of the mentioned metrics.

Values of p < 0.05 were considered statistically significant.

## Results

### Study population

In the descriptive table, it is possible to observe the significant prevalence (p<0.05) of elderly individuals, male and with comorbidities such as hypertension (60.3%), diabetes mellitus (42.2%), and cardiopathy (18.3%) in the severe group compared with the mild group (**Table 1**).

**Table 1:** Characteristics of the population according to the COVID-19 severity and variables included in this study.

Variables/Categories	Mild (n= 216)	%	Severe (n= 292)	%	P-value
Age, Median (IQR)	39.50	(33 - 49)	62	(49 - 75)	**<0.001**
Age > 60 years old	15	6.9%	154	52.7%	**<0.001**
Sex					
Male	71	32.9%	180	61.6%	**<0.001**
Female	145	67.1%	112	38.4%	
Death	0	0%	53	19.7%	**-**
Admission on ICU	0	0%	103	35.9%	–
Comorbidities					
Hypertension	23	10,8%	175	60.3%	**<0.001**
Diabetes mellitus	4	1.9%	122	42.2%	**<0.001^#^**
Obesity	36	17.7%	49	16.9%	0.809
Cardiopathy	1	0.5%	53	18.3%	**<0.001^#^**
COPD	4	1.9%	15	5.2%	0.061^#^
Asthma	12	5.6%	9	3.1%	0.156
Cancer	0	0%	4	1.4%	–

Data are presented as median (IQR). The Mann-Whitney U test was performed for continuous variables (age). The chi-square and Fisher’s exact tests were performed for categorical variables. IQR, interquartile range; ICU, intensive care unit; ^#^Fisher’s exact test.

Bold values indicate statistical significance. P-value < 0.05 is considered significant.

### Description of SNPs in the *AKT1* gene

Two single nucleotide polymorphisms (SNPs) of the *AKT1* gene, rs2494746, and rs1130214, located on chromosome 14, were selected. The rs2494746 is an intronic variant found at position 104791382 (GRCh38.p14; NC_000014.9) that presents a polymorphic G allele with a minor allele frequency (MAF) of 39% in this population. While the rs1130214 variant is located in the 5’ UTR position of the gene, the messenger RNA (mRNA) region that is directly upstream of the initiation codon, with the polymorphic allele A having a MAF of 31% (**Supplementary Table S1**).

### Association of the risk allele *AKT1* rs2494746 with the severity and ICU admission of COVID-19

A significant association was found between the C risk allele of the SNP rs2494746 and the greater chance of the individual presenting the most severe form of the disease after infection with SARS-CoV-2 in the additive model (OR: 1.79; 95%CI: 1.28-2.50) and dominant model (OR: 7.81; 95%CI: 3.74-16.31). The rs1130214 association did not reach statistical significance (**Table 2**).

**Table 2:** A significant association between SNP on *AKT1* gene and severity of COVID-19 by logistic regression adjusted for age, sex, hypertension, diabetes, and cardiopathy.

COVID-19 Severity							
SNP	Model	GENO	Mild n (%)	Severe n (%)	OR	95% CI	P-value
**rs2494746**	ADD	GG	93 (44.5%)	24 (8.6%)	**1.79**	1.28-2.50	**<0.001**
		**CG**	15 (7.2%)	129 (46.2%)			
		**CC**	101 (48.3%)	126 (45.2%)			
	DOM	GG	93 (44.5%)	24 (8.6%)	**7.81**	3.74-16.31	**<0.001**
		**CG** + **CC**	116 (55.5%)	255 (91.4%)			
	REC	GG + **CG**	108 (51.7%)	153 (54.8%)	1.21	0.70-2.06	0.493
		**CC**	101 (48.3%)	126 (45.2%)			
**rs1130214**	ADD	AA	20 (9.5%)	27 (9.4%)	0.99	0.67-1.48	0.975
		**CA**	89 (42.4%)	121 (42.3%)			
		**CC**	101 (48.1%)	138 (48.3%)			
	DOM	AA	20 (9.5%)	27 (9.4%)	1.23	0.50-3.01	0.656
		**CA** + **CC**	190 (90.5%)	259 (90.6%)			
	REC	AA + **CA**	109 (51.9%)	148 (51.7%)	0.92	0.55-1.56	0.764
		**CC**	101 (48.1%)	138 (48.3%)			

SNP, Single Nucleotide Polymorphism; Model, genetic model; GENO, Genotype; OR, Odds Ratio; 95% CI, Confidence Interval; ADD, Additive; DOM, Dominant; REC, Recessive. Bold values ​​indicate statistically significant analyses (P value < 0.05) and emphasize that the C allele was significantly associated with the outcome.

Individuals with severe COVID-19 carrying at least one C allele of the SNP rs2494746 (genotypes CG and CC) exhibited a high frequency of ICU admission. These genotypes were associated with a significantly higher risk of ICU admission, with an OR of 4.026 (95% CI: 1.15-13.98) and a p-value of 0.028, compared to those with the GG genotype (**Table 3**).

**Table 3:** A significant association between SNP in the *AKT1* gene and patients admitted to the ICU with severe COVID-19 by logistic regression adjusted for age, sex, diabetes, and cardiopathy.

COVID-19 ICU							
SNP	Model	GENO	Non-critical n (%)	Critical n (%)	OR	95% CI	P-value
**rs2494746**	ADD	GG	21 (11,79%)	3 (3,061%)	0.990	0.66-1.47	0.961
		**CG**	72 (40,44%)	56 (57,14%)			
		**CC**	85 (47,75%)	39 (39,7%)			
	DOM	GG	21 (11,79%)	3 (3,061%)	**4.026**	1.15-13.98	**0.028**
		**CG** + **CC**	157 (88,20%)	95 (96,93%)			
	REC	GG + **CG**	93 (52,24%)	59 (60,20%)	0.722	0.43-1.19	0.208
		**CC**	85 (47,75%)	39 (39,7%)			
**rs1130214**	ADD	AA	20 (10,98%)	7 (6,930%)	1.047	0.71-1.53	0.814
		**CA**	73 (40,10%)	45 (44,55%)			
		**CC**	89 (48,90%)	49 (48,5%)			
	DOM	AA	20 (10,98%)	7 (6,930%)	1.488	0.60-3.68	0.390
		**CA** + **CC**	162 (89,01%)	94 (93,06%)			
	REC	AA + **CA**	93 (51,09%)	52 (51,48%)	0.933	0.56-1.53	0.784
		**CC**	89 (48,90%)	49 (48,5%)			

SNP, Single Nucleotide Polymorphism; Model, genetic model; GENO, Genotype; OR, Odds Ratio; 95% CI, Confidence Interval; ADD, Additive; DOM, Dominant; REC, Recessive. Bold values ​​indicate statistically significant analyses (P value < 0.05) and emphasize that the C allele was significantly associated with the outcome.

Among all individuals with COVID-19, those carrying at least one C allele of rs2494746 presented more than 7-fold higher chance of being admitted to the ICU compared with patients without this allele-specific expression in the dominant model (OR: 7.81; 95%CI: 3.74-16.31, **Supplementary Table S2**). The rs1130214 was not associated with hospitalization (**Supplementary Table S2**).

### Combining the alleles of rs1130214 and rs2494746 provides an increased risk to worse outcome of COVID-19

The genotype risk score of the SNPs rs2494746 and rs1130214 was analyzed. The individuals were divided into groups without any risk allele or with one risk allele (≤1, GG+AA; GG+AC; AA+CG), with two risk alleles (GG+CC; CC+AA; CG+AC), three risk alleles (CG+CC; CC+AC), and four risk alleles (CC+CC). Individuals with 2 and 3 risk alleles were four times more likely to develop severe COVID-19 (OR:4.04; CI:1.73-9.40; p=0.001 and OR:4.71; IC:2.03-10.97 p<0.001). Meanwhile, individuals with four risk alleles were twice as likely to develop the severity of the disease (OR:2.61; CI:1.21-5.64; p=0.015) (**Table 4**).

**Table 4:** Genotyping risk score analysis for severity, ICU and mortality with rs2494746 and rs1130214 *AKT1* variants.

*AKT1* variants rs2494746 and rs1130214						
Score 1	Risk alleles	Controls (n)	Cases (n)	OR	95% CI	P-value
Severity		Mild	Severe			
	≤1	68	34			
	2	34	73	4.04	1.73-9.40	**0.001**
	3	39	87	4.71	2.03-10.97	**<0.001**
	4	67	83	2.61	1.21-5.64	**0.015**
ICU		Noncritical	Critical			
	≤1	95	7			
	2	75	31	3.62	1.45-9.00	**0.006**
	3	90	34	2.92	1.18-7.22	**0.020**
	4	124	26	1.92	0.77-4.78	0.163
Mortality		Survival	Death			
	≤1	99	2			
	2	83	17	7.8	1.72-35.35	**0.008**
	3	104	16	5.25	1.15-23.9	**0.032**
	4	132	15	4.32	0.95-19.59	0.058

Logistic regression adjusted for age, sex, hypertension, diabetes mellitus and cardiopathy. OR, Odds Ratio; 95% CI, Confidence Interval; P-value <0.05 significant. Bold values ​​indicate statistically significant analyses (P value < 0.05) and emphasize that the C allele was significantly associated with the outcome.

The analysis of disease criticality and mortality demonstrated that individuals with two or three risk alleles were associated with approximately three times greater chance of having the critical condition (OR:3.62; CI:1.45-9.00; p=0.006 and OR:2.92; CI:1.18-7.22; p=0.020) and seven times more chance of dying (OR:7.8; CI:1.72-35.35; p=0.008 and OR:5.25; CI:1.15-23.9; p=0.032) (**Table 4**).

### Genotypic association of rs1130214 and rs2494746 variants with plasma cytokine concentration

**Figure 1:**
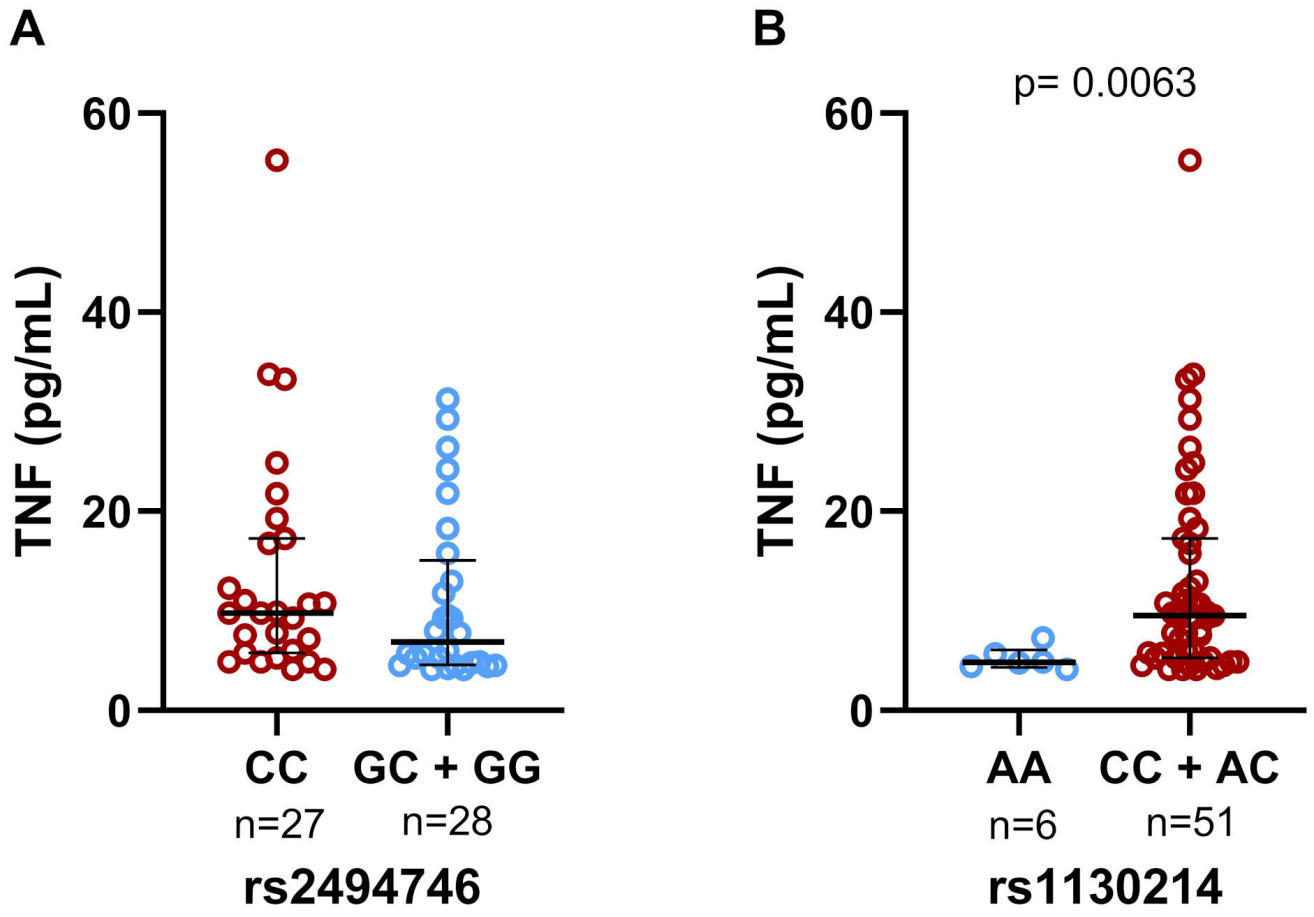
Genotypic association of the rs2494746 and rs1130214 variants with cytokine levels. **(A)** There was no statistical significance in the plasma levels of TNF between the genotypes of the rs2494746; **(B)** The risk allele C (rs1130214) was significantly associated with increased levels of TNF. Data are presented as median and interquartile ranges for non-parametric distribution. The unpaired two-tailed Mann-Whitney U test was used for comparisons between groups (p= 0.0063).

Individuals homozygous AA of rs1130214 had low concentrations of TNF when compared with individuals who carried the allele C (AC/CC) (p =0.0063, **Figure 1B**). We did not find statistical significance when analyzing the association between TNF levels and rs2494746 genotypes (**Figure 1A**). No difference was found in IL-6 and CCL2 levels between the genotypes of both SNPs (**Supplementary Figure S1**).

Analyzing the cytokine levels and the genetic profiles of individuals afflicted with severe COVID-19, we noted findings consistent with our prior study. Notably, individuals carrying the rs1130214 risk allele (C) exhibited elevated concentrations of TNF. (p=0.0014, **Supplementary Figure 2B**).

### Genotypic association of rs1130214 and rs2494746 variants with blood D-dimer concentration

**Figure 2:**
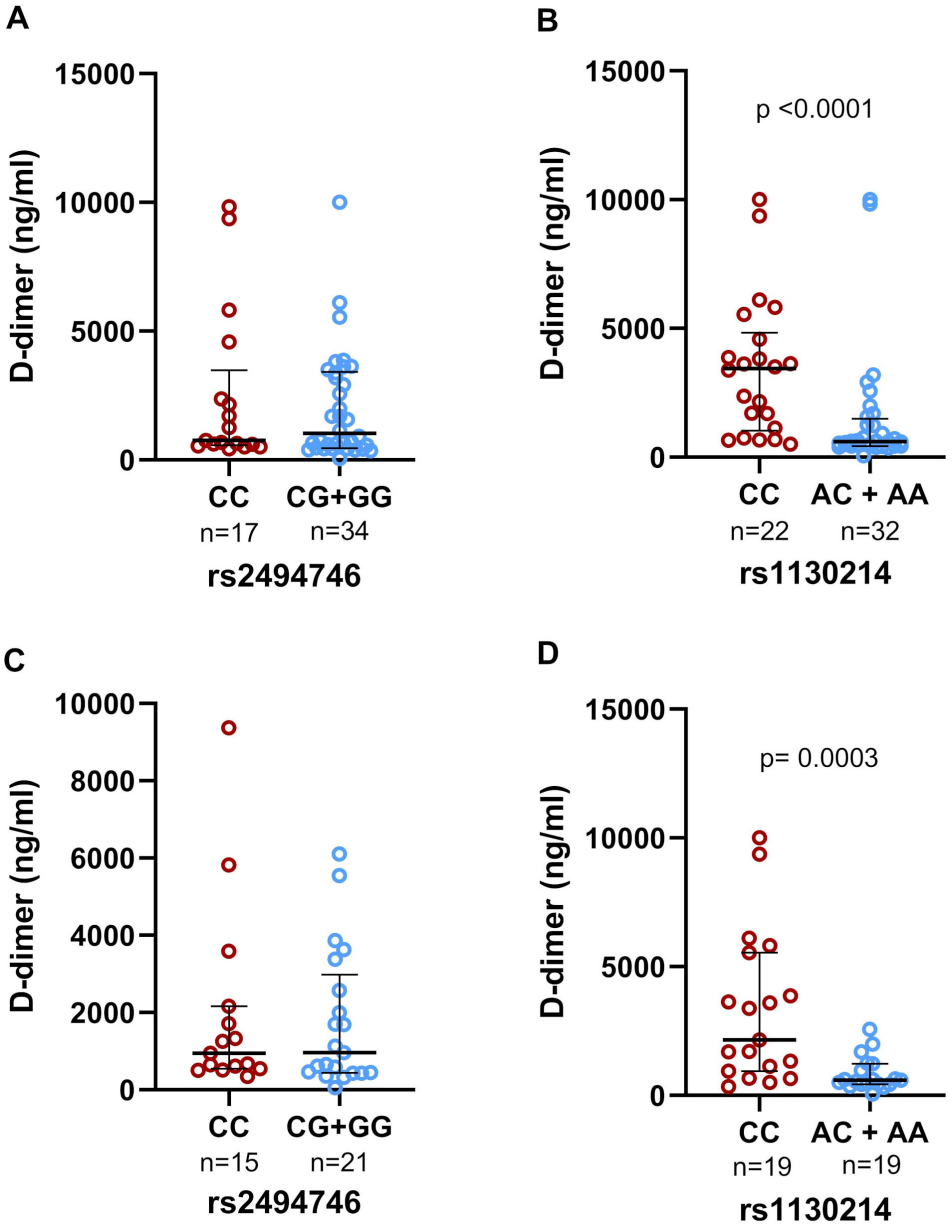
Genotypic association of the rs2494746 and rs1130214 variants with d-dimer levels in the plasma of individuals with COVID-19 in severe cases of Paraíba. There was no statistical difference between the rs2494746 genotypes in **(A)** individuals admitted to the ICU and **(C)** individuals who died. Homozygotes for the C risk allele (rs1130214) were significantly associated with increased d-dimer levels compared to individuals carrying the AC or AA genotype in **(B)** individuals admitted to the ICU and **(D)** individuals who died. Data are presented as median and interquartile ranges for non-parametric distribution. The unpaired two-tailed Mann-Whitney U test was used for comparisons between groups (p<0.0001 – p=0.0003).

When analyzing D-dimer levels and genotypes among patients with COVID-19 admitted to the ICU, we observed that rs1130214 homozygous CC individuals had high concentrations of D-dimer when compared to individuals with the A allele (AA/AC) (p<0.0001, **Figure 2B**). This difference was not observed in rs2494746 (**Figure 2A**).

Furthermore, when analyzing the D-dimer concentration in patients who died, individuals with genotype CC of rs1130214 had higher concentrations than AA/AC individuals (p=0.0003, **Figure 2D**). The rs2494746 variant showed no significant difference (**Figure 2C**).

### Survival analysis in patients with severe COVID-19 with rs1130214 and rs2494746 variants

**Figure 3:**
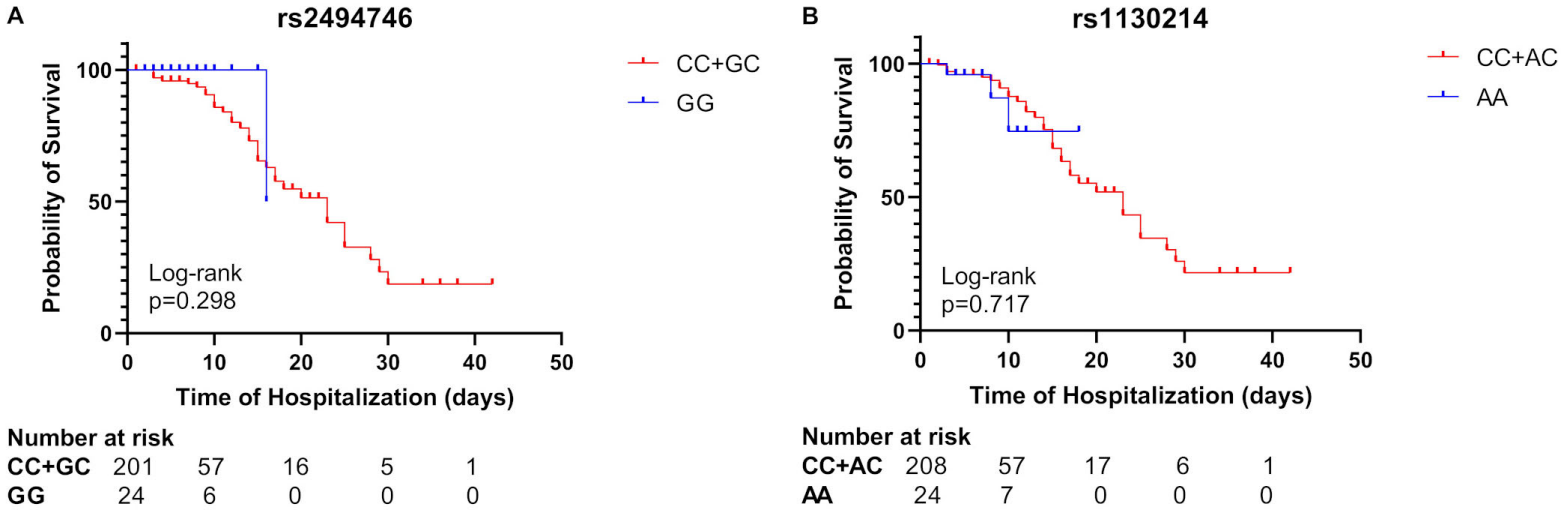
Kaplan–Meier survival curves of severe COVID-19 patients for genotype of rs2494746 and rs1130214 *AKT1* variants. The recessive models showed no statistically significant difference between individuals with at least one C allele compared to homozygote individuals in the rs2494746 variant **(A)** or the rs1130214 **(B)**.

The Kaplan-Meier graph showed no significant difference in the survival of individuals infected with SARS-CoV-2 based on different genotypes of the studied variants. However, patients with at least one C allele of *AKT1* variants exhibited a prolonged hospital stay compared to homozygote individuals (**Figures 3A, B**). This suggests that the presence of the C allele may be associated with an extended duration of hospitalization and an increased risk of mortality from COVID-19.

Regarding the relationship between rs2494746-C allele and mortality, we observed 12.86-fold increased COVID-19 mortality compared with subjects without C allele at rs2494746 in the dominant model (p=0.013, **Supplementary Table S3**). No significant association with mortality was found for rs1130214 (**Supplementary Table S3**).

### Predictive analysis of risk alleles with COVID-19 severity

In the predictive analysis for rs2494746, with 494 individuals, we obtained a sensitivity of 91% and specificity of 45%, a positive predictive value of 69% and a negative predictive value of 80% (Kappa concordance index= 0.384, accuracy=71% and p<0.001). The rs1130214, with 502 individuals, had a sensitivity of 90% and specificity of 10%, a positive predictive value of 58% and a negative predictive value of 43% (Kappa concordance index= 0.002). In addition, it presents an accuracy of 56% and p>0.05, demonstrating that it is not statistically significant or accurate (**Table 5**).

**Table 5:** Prediction analysis for severity with rs2494746 and rs1130214 *AKT1* variants.

COVID-19 Severity								
	Allele (n)		Test accuracy indices (%)					
	Risk	Polymorphic	Accuracy	Sensitivity	Specificity	^a^ PPV	^b^ NPV	P-value
**rs2494746**	CC + CG	GG						
Cases	258	117	**71**	**91**	**45**	**69**	**80**	**<0.001**
Controls	24	95						
**rs1130214**	CC + AC	AA						
Cases	261	192	56	90	10	58	43	0.949
Controls	28	21						

a PPV, Positive predictive value;

b NPV, Negative predictive value; P-value <0.05 significant. Bold values ​​indicate statistically significant analyses (P value < 0.05) and emphasize that the C allele was significantly associated with the outcome.

## Discussion

The study analyzed the association between the rs1130214 and rs2494746 variants of the *AKT1* gene with the severity of COVID-19. We found that individuals who presented the C allele of rs2494746 are eight times more likely to develop severe COVID-19 and require ICU support.

This is the first study that correlates variants on the *AKT1* gene with COVID-19. A previous study associated rs2494746 with an increased risk of developing type 2 diabetes mellitus (11). COVID-19 has also been associated with an increased risk of developing new-onset diabetes after infection (16, 17), suggesting that COVID-19 and diabetes share a mechanism of genetic predisposition.

In silico functional analysis from Regulome Db 2.2 uses two distinct metrics, ranking and scoring, to assess the relevance and reliability of genetic variants in genomic studies. The rs2494746 variant received a rank of 1f, indicating strong evidence of a functional effect, possibly related to gene regulation or other biological functions. The score assigned to the variant (0.22271) reflects the confidence in the classification, based on several functional characteristics (**Supplementary Table S1**). In summary, the results suggest that the rs2494746 variant plays a significant biological role, with strong evidence for a functional effect, possibly involved in gene regulation or other relevant biological processes. Furthermore, in the lungs, this variant is associated with strong transcriptional activity and acts as an enhancer of gene expression in genetic regulatory elements, potentially influencing lung function and playing a crucial role in the immune response to respiratory infections.

SARS-Cov-2 binds to the ACE2 receptor and activates many signaling pathways promoting the production of inflammatory cytokines, including TNF, IL-17, IL-6, IL-8, and IL-1β. These cytokines are secreted by numerous cells, such as epithelial cells, macrophages, T lymphocytes, neutrophils, and Th17 cells, and exacerbate SARS-CoV-2 infection. Excessive release of cytokines has consequences such as acute respiratory distress syndrome (ARDS) (2).

The PI3K/AKT pathway plays a crucial role in regulating various cellular functions such as proliferation, survival, cell cycle, apoptosis, glycogen metabolism, and inflammation. It can modulate the synthesis of pro-inflammatory cytokines (IL-1β, IL-6, and IL-8) induced by TNF (18), trigger TGF-β1 activation and protein phosphorylation, leading to excessive proliferation and differentiation of fibroblasts in the lungs (19). Therefore, genetic variants on *AKT* can play a key role in the production of cytokines (20) and may be related to an exacerbated immune response to COVID-19.

According to the HaploReg v4.2 platform, the rs1130214 variant, located in the 5’-UTR region of the *AKT1* gene, is associated with regulatory elements such as protein binding sites and altered transcription motifs in various cells and tissues, including T cells, B cells and peripheral blood mononuclear cells. The presence of these regulatory elements suggests that the variant can influence gene activity in cells involved in regulating the inflammatory response and the production of cytokines, such as TNF. Furthermore, the presence of binding sites for known transcription factors, such as CTCF and RAD21, indicates that the variant may be involved in chromatin organization and transcription regulation (21, 22). This information highlights the importance of the rs1130214 variant in regulating gene expression and modulating biological processes associated with the *AKT1* gene.

We also observed an association between the CC genotype of the rs1130214 variant and higher D-dimer levels compared to AA/AC. As a coagulation marker, increased D-dimer reflects hypercoagulability and thrombotic burden. Higher D-dimer levels have been reported to provide useful prognostic information regarding COVID-19 progression and mortality (23). It is known that AKT phosphorylation is involved in thrombus formation through the PI3K/AKT signaling pathway that activates platelets, and the use of PI3K or AKT inhibitors potently abolishes the increased binding of platelet fibrinogen induced by incubation with patient sera with COVID-19 admitted to the ICU (24). This suggests a possible contribution of this variant to thrombotic events in COVID-19.

Here, individuals with the risk allele (C) of the rs1130214 were associated with increased TNF production compared to the AA genotype. Furthermore, the CC genotype of rs1130214 was associated with higher levels of D-dimer. These results suggest a contribution of this variant in the cytokine storm and blood clotting. The rs1130214 has been associated with persistently high CD4+ T cell glycolytic activity in HIV-positive individuals (10), increased risk of having congenital heart disease (12), glucose homeostasis and metabolic syndrome (9), among other conditions.

Genotype risk score analysis revealed a cumulative effect of the risk alleles of the rs1130214 and rs2494746 variants with a greater chance of severity, criticality, and mortality of COVID-19. This finding supports the hypothesis that the co-inheritance of these variants may heighten an individual’s susceptibility to severe COVID-19. However, the specific molecular mechanisms underlying this phenomenon remain unclear.

The predictive power evaluation of rs2494746 revealed an accuracy of 71%, suggesting that such variant may be a valuable marker to assist in the early detection of individuals who will develop severe COVID-19 following SARS-CoV-2 infection. Other studies demonstrated that serum levels of IL-6 and TNF remained independent and significant predictors of disease severity and death (25), identified a panel of laboratory indicators of severe disease, such as creatinine levels, neutrophil count, C-reactive protein (CRP) and D-dimer (26). In addition to biomarkers to assess the risk of thrombosis in these patients, including markers of platelet activation, platelet aggregation, activation or injury of endothelial cells, coagulation, and fibrinolysis (27). Although a panel of laboratory indicators of severe disease has been identified, these values represent relatively nonspecific markers of an increased inflammatory state and organ dysfunction. Consequently, this variant has the potential to be integrated into the existing set of clinical markers, enhancing the precision of disease prognosis.

Nonetheless, it is essential to acknowledge certain limitations inherent to this study. First, the GG genotype of the rs2494746 variant was detected in a relatively small proportion of ICU patients who ultimately died, and this observation may have had an impact on our findings. Second, this study was unpowered to demonstrate whether the variants alter gene expression, and the predictive values may not be confirmed in the current population. Despite these restrictions, this sample is made up of unvaccinated people. This has a positive point, which is the original response to the virus, thus improving the understanding of pathogenic mechanisms. This study is pioneering in highlighting the impact of genetic variants of the *AKT1* gene on the more unfavorable prognosis of COVID-19.

In conclusion, the rs2494746 variant was associated with risk of severity and critical outcome, as well as death from COVID-19. Meanwhile, rs1130214 was related to increased levels of TNF and D-dimer. These variants presented cumulative risk when inherited together. Furthermore, the predictive analysis demonstrated a moderate probability of the individual with the risk allele of the rs2494746 variant developing the severity of the disease. Therefore, the present study contributes to understanding the influence of the *AKT1* gene and its variants on the immunological damage in individuals infected with SARS-CoV-2. This could be useful in the future to assist in predicting a worse outcome of COVID-19.

## Data Availability

The datasets presented in this study can be found in online repositories. The names of the repository/repositories and accession number(s) can be found below: https://www.ncbi.nlm.nih.gov/snp/, rs2494746; https://www.ncbi.nlm.nih.gov/snp/, rs1130214.
