# Supplementary material for: Genetic signatures of AKT1 variants associated with worse COVID-19 outcomes – a multicentric observational study
Source: Front Immunol. 2024 Oct 8;15:1422349. doi: 10.3389/fimmu.2024.1422349 (PMC11493623; doi:10.3389/fimmu.2024.1422349)
Supplement: Supplementary file 1 [file DataSheet1.docx]

**Supporting Information**

**Supplementary Table S1.** Description of SNPs in the *AKT1* gene.

| **CHR** | **SNP** | **Position** | **Alleles^*^** | **MAF** | **Function^#^** | **Regulome Db**  **Rank (score)** |
| --- | --- | --- | --- | --- | --- | --- |
| 14 | rs2494746 | 104791382 | **G**/C | 0.39 | Intron variant | 1f (0.22271) |
| 14 | rs1130214 | 104793397 | **A**/C | 0.31 | 5 prime UTR variant | 1f (0.55436) |

^*^ Polymorphic allele/Reference allele; ^#^ Ensembl’s function; CHR: Chromosome; MAF: Minor allele frequency.

**Supplementary Table S2.** A significant association between SNP in the *AKT1* gene and patients admitted to the ICU with COVID-19 by logistic regression adjusted for age, sex, diabetes, and cardiopathy.

| **COVID-19 ICU** | | | | | | | |
| --- | --- | --- | --- | --- | --- | --- | --- |
| **SNP** | **Model** | **GENO** | **No admission**  **n (%)** | **ICU- admission**  **n (%)** | **OR** | **95% CI** | **P value** |
| **rs2494746** | ADD | GG | 114 (29.5%) | 3 (3.1%) | 1.16 | 0.83-1.62 | 0.374 |
|  |  | **CG** | 87 (22.5%) | 56 (57.1%) |  |  |  |
|  |  | **CC** | 186 (48.1%) | 39 (39.8%) |  |  |  |
|  | DOM | GG | 114 (29.5%) | 3 (3.1%) | **7.74** | 2.35-25.47 | **<0.001** |
|  |  | **CG** + **CC** | 273 (70.5%) | 95 (96.9%) |  |  |  |
|  | REC | GG + **CG** | 201 (51.9%) | 59 (60.2%) | 0.68 | 0.42-1.11 | 0.126 |
|  |  | **CC** | 186 (48.1%) | 39 (39.8%) |  |  |  |
| **rs1130214** | ADD | AA | 40 (10.2%) | 7 (6.9%) | 1.02 | 0.71-1.46 | 0.933 |
|  |  | **CA** | 162 (41.3%) | 45 (44.6%) |  |  |  |
|  |  | **CC** | 190 (48.5%) | 49 (48.5%) |  |  |  |
|  | DOM | AA | 40 (10.2%) | 7 (6.9%) | 1.37 | 0.57-3.30 | 0.482 |
|  |  | **CA** + **CC** | 352 (89.8%) | 94 (93.1%) |  |  |  |
|  | REC | AA + **CA** | 202 (51.5%) | 52 (51.5%) | 0.93 | 0.58-1.50 | 0.763 |
|  |  | **CC** | 190 (48.5%) | 49 (48.5%) |  |  |  |

SNP: Single Nucleotide Polymorphism; Model: genetic model; GENO: Genotype; OR: Odds Ratio; 95% CI: Confidence Interval; ADD: Additive; DOM: Dominant; REC: Recessive.

**Supplementary Table S3.** A Significant association between SNP on *AKT1* gene and mortality of COVID-19 by logistic regression adjusted for age and sex.

| **COVID-19 Mortality** | | | | | | | |
| --- | --- | --- | --- | --- | --- | --- | --- |
| **SNP** | **Model** | **GENO** | **Survival**  **n (%)** | **Death**  **n (%)** | **OR** | **95% CI** | **P value** |
| **rs2494746** | ADD | GG | 118 (28.0%) | 1 (2.0%) | 1.32 | 0.87-2.02 | 0.197 |
|  |  | **CG** | 104 (24.7%) | 27 (54.0%) |  |  |  |
|  |  | **CC** | 199 (47.3%) | 22 (44.0%) |  |  |  |
|  | DOM | GG | 118 (28.0%) | 1 (2.0%) | **12.86** | 1.73-95.59 | **0.013** |
|  |  | **CG** + **CC** | 303 (72.0%) | 49 (98.0%) |  |  |  |
|  | REC | GG + **CG** | 222 (52.7%) | 28 (56.0%) | 0.88 | 0.48-1.62 | 0.684 |
|  |  | **CC** | 199 (47.3%) | 22 (44.0%) |  |  |  |
| **rs1130214** | ADD | AA | 43 (10.1%) | 3 (5.8%) | 1.09 | 0.68-1.73 | 0.720 |
|  |  | **CA** | 176 (41.2%) | 23 (44.2%) |  |  |  |
|  |  | **CC** | 208 (48.7%) | 26 (50.0%) |  |  |  |
|  | DOM | AA | 43 (10.1%) | 3 (5.8%) | 1.78 | 0.52-6.12 | 0.362 |
|  |  | **CA** + **CC** | 384 (89.9%) | 49 (94.2%) |  |  |  |
|  | REC | AA + **CA** | 219 (51.3%) | 26 (50.0%) | 0.98 | 0.54-1.78 | 0.947 |
|  |  | **CC** | 208 (48.7%) | 26 (50.0%) |  |  |  |

SNP: Single Nucleotide Polymorphism; Model: genetic model; GENO: Genotype; OR: Odds Ratio; 95% CI: Confidence Interval.

### Supplementary Figure S1. Genotypic association of *AKT1* gene variants and cytokine levels in patients infected with SARS-CoV-2. We found no significant difference in plasma IL-6 concentration between rs2494746 (A) and rs1130214 (B) genotypes. No statistically significant difference was found in the plasma concentration of CCL2 either (C-D). Mann Whitney U test p >0.05.

### Supplementary Figure S2. Genotypic association of *AKT1* gene variants and cytokine levels in severe COVID-19. The risk allele C (rs1130214) was significantly associated with increased levels of TNF (B); We found no significant difference in the plasma concentration of TNF between the genotypes of rs2494746 (A); There was no statistical significance in the plasmatic levels of IL-6 or CCL2 between the genotypes of rs1130214 (D-F) and rs2494746 (C-E). Mann-Whitney U test.
