# Supplementary figures and images for: Genetic signatures of AKT1 variants associated with worse COVID-19 outcomes – a multicentric observational study
Source: Front Immunol. 2024 Oct 8;15:1422349. doi: 10.3389/fimmu.2024.1422349 (PMC11493623; doi:10.3389/fimmu.2024.1422349)

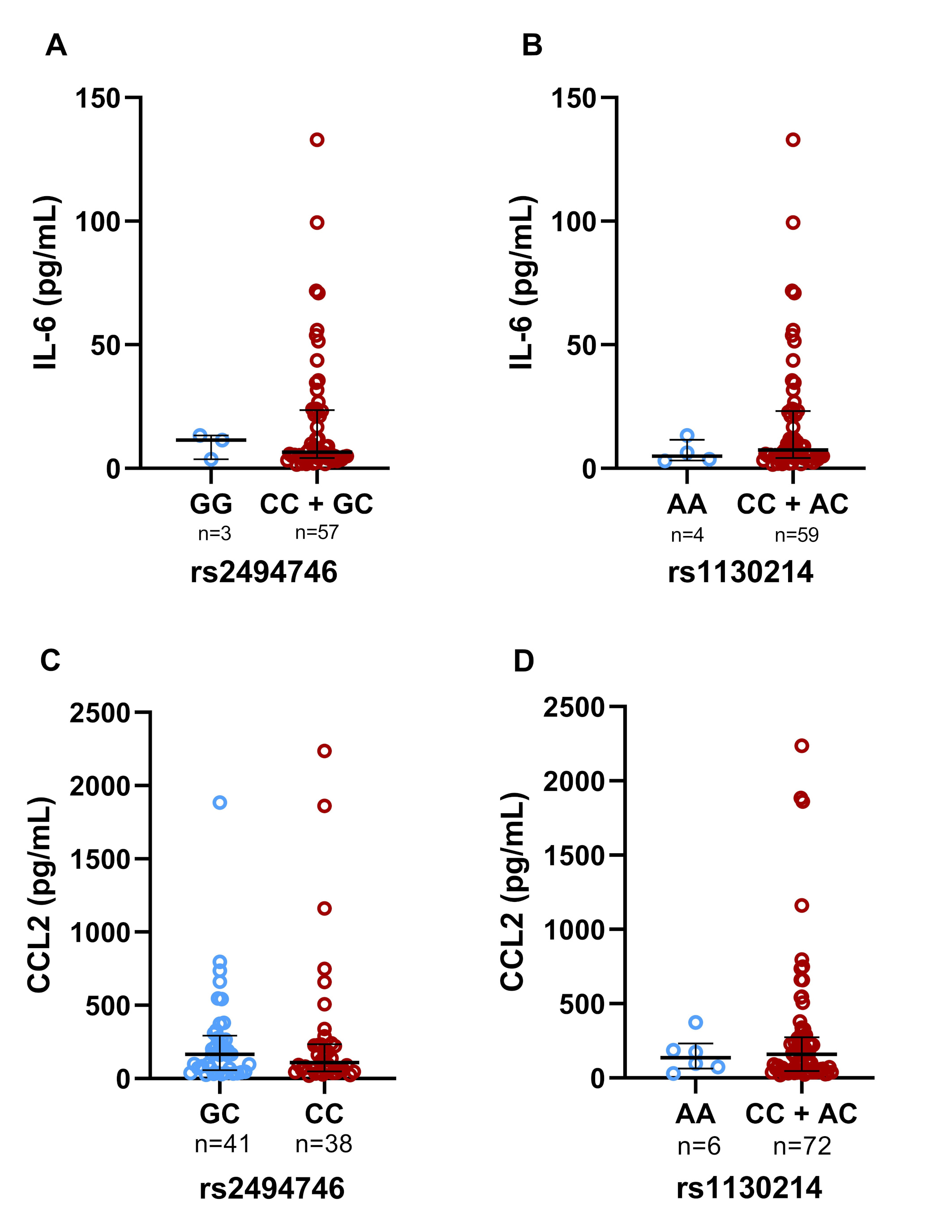

Supplement: Supplementary file 2 [file Image1.jpeg]

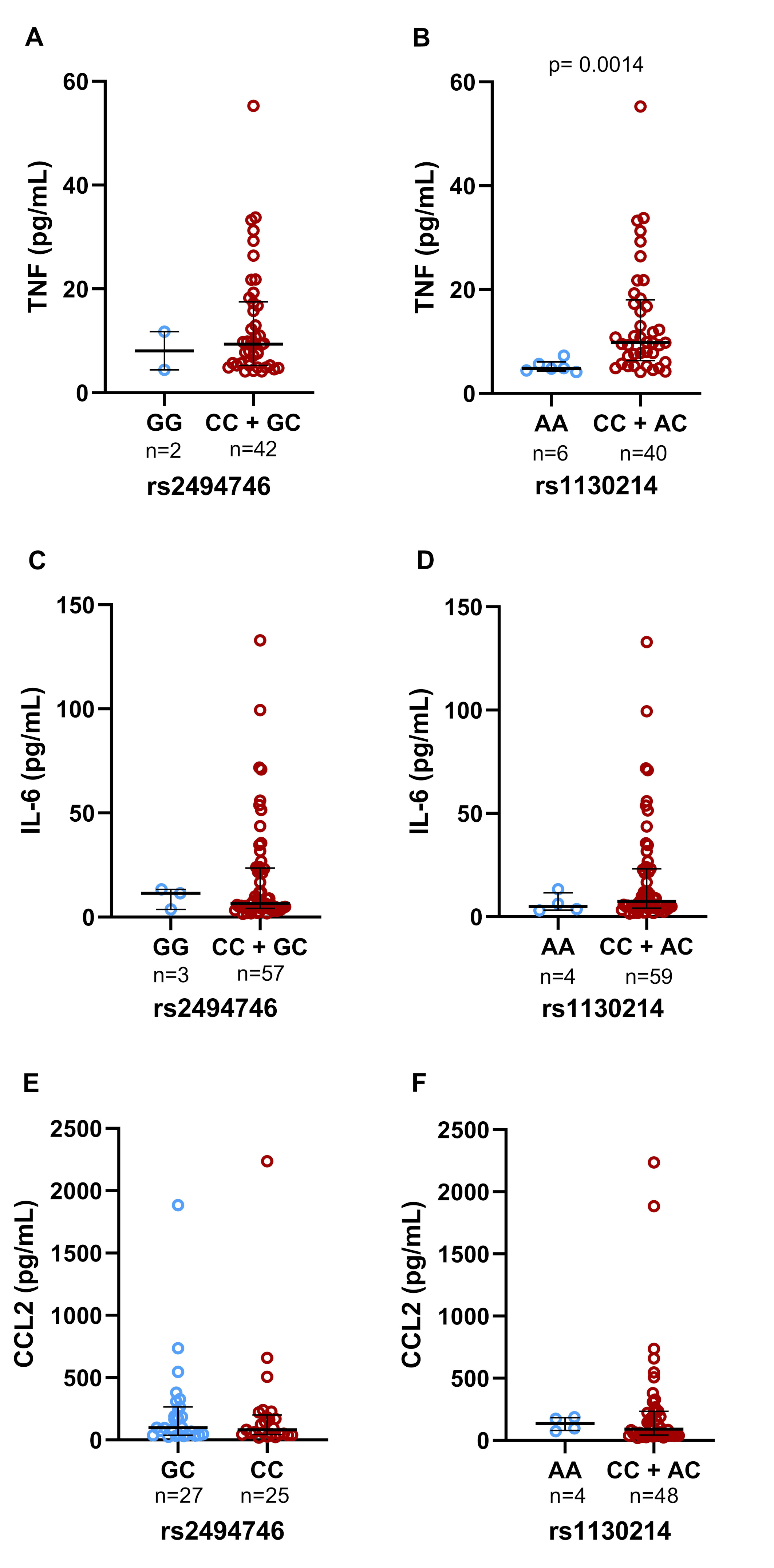

Supplement: Supplementary file 3 [file Image2.jpeg]
